# CD38 ligation in sepsis promotes nicotinamide phosphoribosyltransferase-mediated IL-6 production in kidney stromal cells

**DOI:** 10.1093/ndt/gfae269

**Published:** 2024-11-20

**Authors:** Yuya Suzuki, Tadashi Otsuka, Yusuke Takahashi, Shingo Maruyama, Alexey Annenkov, Yasuhiro Kanda, Tomoya Katakai, Hirofumi Watanabe, Riuko Ohashi, Yoshikatsu Kaneko, Ichiei Narita

**Affiliations:** Division of Clinical Nephrology and Rheumatology, Kidney Research Center, Niigata University Graduate School of Medical and Dental Sciences, Niigata, Japan; Division of Clinical Nephrology and Rheumatology, Kidney Research Center, Niigata University Graduate School of Medical and Dental Sciences, Niigata, Japan; Division of Clinical Nephrology and Rheumatology, Kidney Research Center, Niigata University Graduate School of Medical and Dental Sciences, Niigata, Japan; Division of Clinical Nephrology and Rheumatology, Kidney Research Center, Niigata University Graduate School of Medical and Dental Sciences, Niigata, Japan; Histopathology Core Facility, Faculty of Medicine, Niigata University, Niigata, Japan; Department of Immunology, Niigata University Graduate School of Medical and Dental Sciences, Niigata, Japan; Department of Immunology, Niigata University Graduate School of Medical and Dental Sciences, Niigata, Japan; Division of Clinical Nephrology and Rheumatology, Kidney Research Center, Niigata University Graduate School of Medical and Dental Sciences, Niigata, Japan; Histopathology Core Facility, Faculty of Medicine, Niigata University, Niigata, Japan; Division of Clinical Nephrology and Rheumatology, Kidney Research Center, Niigata University Graduate School of Medical and Dental Sciences, Niigata, Japan; Division of Clinical Nephrology and Rheumatology, Kidney Research Center, Niigata University Graduate School of Medical and Dental Sciences, Niigata, Japan

**Keywords:** AKI, IL-6, NAMPT, renal stromal cell, sepsis

## Abstract

**Background:**

Activated macrophages, pivotal for driving the immune response in sepsis, express high levels of CD38. Although the circulating levels of its ligand, CD31, increase in sepsis, the functions of CD38 and its ligation remain elusive. This study aimed to elucidate the impact of CD38 ligation on sepsis using single-cell and single-nucleus RNA sequencing (scRNA-seq and snRNA-seq, respectively) to identify a novel therapeutic target for severe sepsis.

**Methods:**

We performed scRNA-seq analysis of mouse peritoneal immune cells to precisely identify cell types exhibiting increased CD38 expression upon exposure to lipopolysaccharide (LPS). Subsequently we induced CD38 ligation using a well-established agonistic anti-CD38 antibody in a mouse model of LPS-induced sepsis. We analysed its pathophysiological effects using kidney snRNA-seq. Finally, we performed histological analysis of septic tissues collected from patients to ensure consistency of our findings between mice and humans.

**Results:**

LPS stimulation upregulated CD38 expression in peritoneal macrophages. CD38 ligation significantly exacerbated LPS-induced inflammation *in vivo*, particularly in the kidneys. Kidney snRNA-seq analysis revealed that CD38 ligation induced interleukin (IL)-6 production in renal stromal cells via nicotinamide phosphoribosyltransferase (NAMPT) signalling originating from CD38-positive macrophages. NAMPT inhibition significantly ameliorated LPS-induced IL-6 production and kidney injury. Histological analysis of human septic tissues demonstrated upregulation of *IL6* messenger RNA and NAMPT in renal stromal cells and CD38-positive macrophages, respectively.

**Conclusion:**

Our findings elucidate the implications of CD38 ligation in an LPS-induced sepsis model and uncover shared signalling pathways between mice and human sepsis. NAMPT signalling identified in this study may be a novel therapeutic target for mitigating systemic inflammation and kidney injury associated with severe sepsis.

## Graphical Abstract

**Graphical Abstract:**
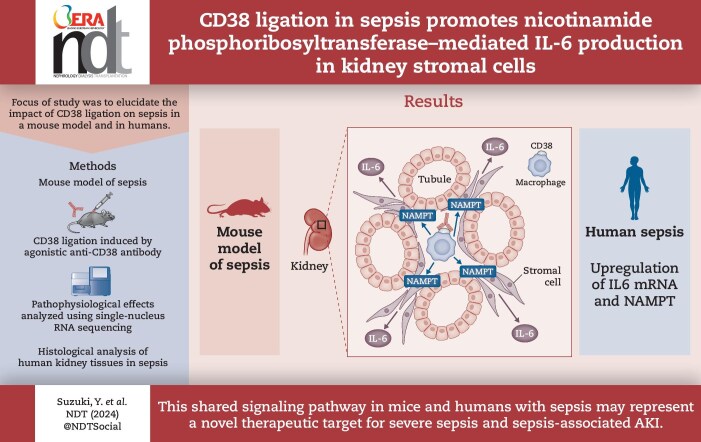

KEY LEARNING POINTS **What was known:**

- Despite recent advancements in critical care, the mortality rate associated with septic shock remains high.
- Activated macrophages, pivotal for driving immune responses in sepsis, express high levels of CD38.
- Although the circulating levels of its ligand, CD31, also increase in sepsis, the functions of CD38 and its ligation remain elusive.

**This study adds:**

- CD38 ligation exacerbated LPS-induced inflammation *in vivo*, particularly in the kidneys.
- Kidney snRNA-seq analysis revealed that CD38 ligation induced interleukin-6 production in renal stromal cells via nicotinamide phosphoribosyltransferase (NAMPT) signalling originating from CD38-positive macrophages.
- Histologically, human septic tissues demonstrated *IL6* messenger RNA and NAMPT upregulation in renal stromal cells and CD38-positive macrophages, respectively.

**Potential impact:**

- Our findings elucidate the implications of CD38 ligation in a lipopolysaccharide-induced sepsis model and uncover shared signalling pathways between mice and human sepsis.
- NAMPT signalling identified in this study may be a novel therapeutic target for the treatment of systemic inflammation and kidney injury induced by severe sepsis.

## INTRODUCTION

Sepsis is a life-threatening organ dysfunction caused by dysregulated immune responses to infections [1]. Although inflammatory cytokines are pivotal in regulating immune responses, their excessive production correlates strongly with sepsis exacerbation [1, 2]. The serum levels of tumour necrosis factor (TNF)-α, interleukin (IL)-1β and IL-6 are strongly associated with mortality [3]. However, as the blocking of these cytokines fails to reduce overall mortality due to sepsis [4–6], unknown cell–cell signalling pathways may be associated with these cytokines. Despite recent advancements in critical care, the mortality rate associated with septic shock has not improved [7].

Macrophages play a pivotal role in the immune response during sepsis [8, 9]. In the initial stage of sepsis, pro-inflammatory factors, such as lipopolysaccharide (LPS), trigger the polarization of classically activated macrophages, leading to the production of inflammatory cytokines [10]. Compared with other phenotypes, classically activated macrophages express high levels of CD38 [11–13], a transmembrane protein involved in nicotinamide adenine dinucleotide (NAD) metabolism [14]. However, the role of CD38 in either protecting against or exacerbating sepsis-induced inflammation remains controversial. Previous studies using CD38 knockout mice have demonstrated amelioration of sepsis-induced neuroinflammation [15] and exacerbation of lung [16, 17], liver [18, 19] and kidney injury [20]. Considering that the genetic knockout of CD38 reportedly induces compensatory enhancement of Toll-like receptor 4 [19, 20], these results may be influenced by changes in other signalling pathways. In contrast, pharmacological inhibition of CD38 has been shown to ameliorate neuroinflammation [15] and kidney injury [11]. However, the flavonoids used as CD38 inhibitors in these studies also exert antioxidant and anti-inflammatory effects [21, 22] and may directly influence the pathology of sepsis. Therefore, the role of CD38 in sepsis remains unclear.

Additionally, the circulating levels of CD31, a well-known ligand of CD38 [23], also increase in patients with sepsis [24]. Although several *in vitro* studies have reported that CD38 ligation induces cytokine production and cell adhesion in macrophages and monocytes [25, 26], studies investigating the effects of CD38 ligation in *in vivo* sepsis models are lacking. As the development of sepsis is mediated by complicated cell–cell interactions via cytokines between various cell types, the impact of CD38 ligation on sepsis needs to be investigated *in vivo* and in individual cells to clarify the alterations in cell–cell interactions.

Based on the above, this study aimed to elucidate the impact of CD38 ligation on sepsis using single-cell and single-nucleus RNA sequencing (scRNA-seq and snRNA-seq, respectively) analyses. Our findings may help identify a novel therapeutic target for severe sepsis.

## MATERIALS AND METHODS

Details can be found in the supplementary data.

### Study design

This study aimed to elucidate the impact of CD38 ligation on sepsis. First, we collected peritoneal immune cells from 8- to 12-week-old male C57BL/6J mice and stimulated them with 100 ng/ml LPS from *Escherichia*  *coli* O127:B8 (Sigma-Aldrich, St. Louis, MO, USA) to identify the cell types that increase CD38 expression through scRNA-seq analysis. Second, we injected 10 mg/kg LPS into male C57BL/6J mice (age 8–12 weeks) to investigate CD38 expression in each organ via reverse transcription quantitative polymerase chain reaction (RT-qPCR) analysis. Third, CD38 ligation was achieved in this LPS-induced sepsis model by administering 50 mg/kg of well-established agonistic anti-CD38 (clone NIMR-5) [27, 28], then its impact was investigated using kidney snRNA-seq analysis. Finally, we obtained human samples from autopsy or kidney biopsy samples previously collected and stored at Niigata University and performed immunostaining and *in situ* hybridization (ISH) in the tissues of patients with sepsis or healthy donors to ensure the consistency of our findings between mice and humans. The sample size for each experimental group was determined based on our experience with similar studies. Consistent results obtained from more than two technical replicates per experiment were used herein.

### Statistical analysis

Statistical analyses were performed using GraphPad Prism version 9.2.0 (GraphPad Software, San Diego, CA, USA). Normally distributed data were expressed as mean ± standard deviation, whereas non-normally distributed data were presented as medians (interquartile ranges). For normally distributed data, statistical significance was determined using Student's two-tailed *t*-test or one-way analysis of variance with Tukey's multiple comparison test. For non-normally distributed data, the Wilcoxon signed-rank test or the Kruskal–Wallis test with Dunn's multiple comparison test was used. The significance levels were denoted as follows: *P < .05, **P < .01, ***P < .001 and ****P < .0001.

## RESULTS

### *Cd38* was upregulated in mouse macrophages in response to LPS stimulation

**Figure 1:**
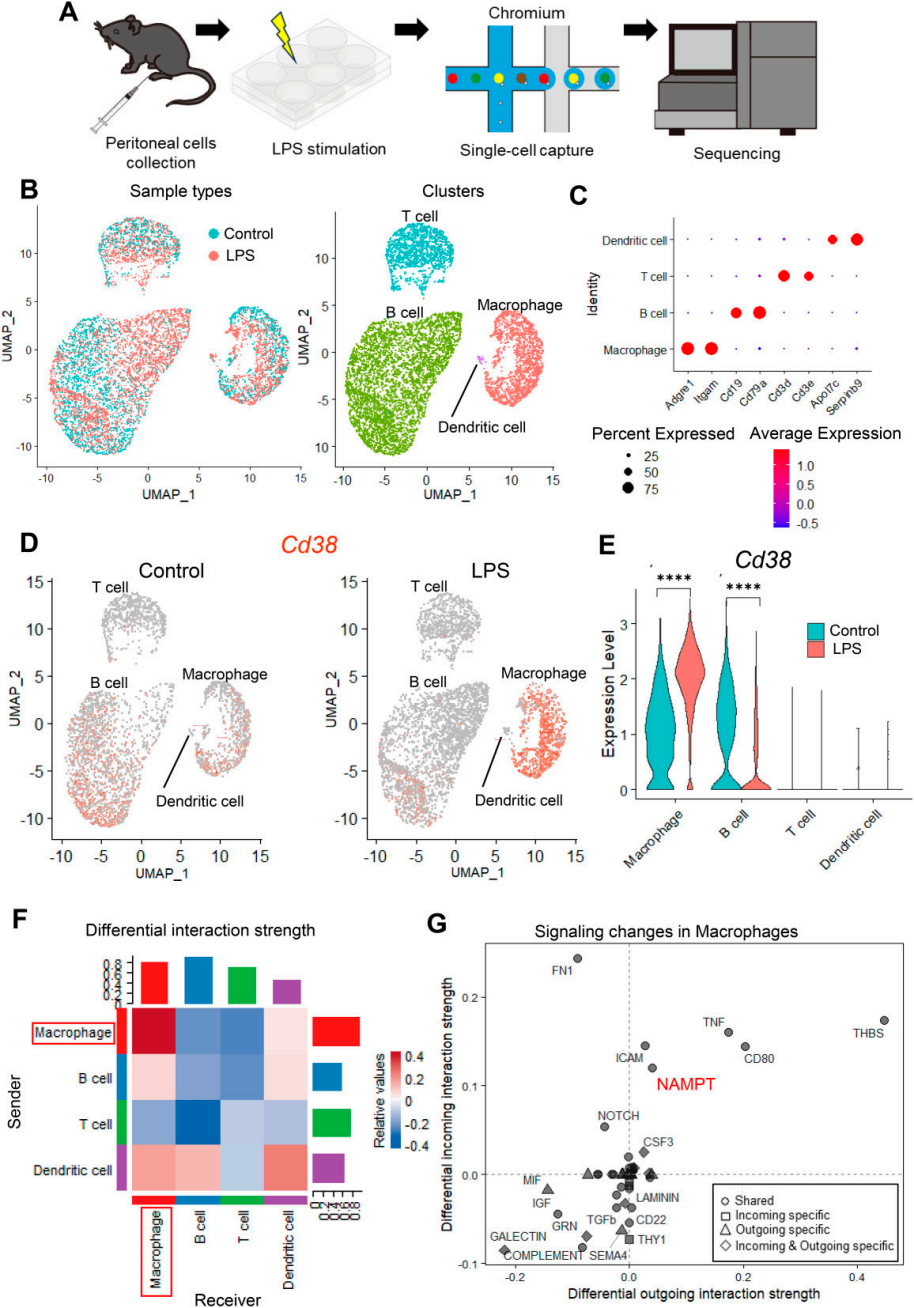
*Cd38* was upregulated in mouse macrophages in response to LPS stimulation. **(A)** Schematic representation of the scRNA-seq protocol. **(B)** Uniform manifold approximation and projection plot displaying cell clusters. **(C)** Dot plot presenting the expression of cell type–specific markers used for cluster identification. **(D)** Feature plot and **(E)** violin plot illustrating *Cd38* expression. **(F)** Heat map showing the differential interaction strength in the cell–cell communication network. **(G)** Scatter plot showing specific signalling changes in macrophages induced by LPS stimulation.

To identify the cell types expressing *Cd38* upon LPS stimulation, we performed scRNA-seq analysis of mouse peritoneal immune cells (Fig. 1A). First, we assessed the temporal upregulation of *Cd38* induced by LPS using RT-qPCR (Supplementary Fig. S1A) and performed scRNA-seq 4 hours after LPS exposure. Four cell types, including macrophages, B cells, T cells and dendritic cells, were identified (Fig. 1B and C) and significant upregulation of innate immunity–related genes was observed in each cell cluster following LPS stimulation (Supplementary Fig. S2). Although the macrophages and B cells predominantly expressed *Cd38* in the absence of LPS, the macrophages exhibited upregulation of *Cd38* following LPS exposure, whereas the B cells showed downregulation (Fig. 1D and E). As peritoneal macrophages cultured with the protein synthesis inhibitor cycloheximide also exhibited *Cd38* mRNA upregulation in response to LPS, it is plausible that CD38 upregulation in macrophages represents a direct effect of LPS stimulation rather than a secondary response (Supplementary Fig. S1B). Further, we investigated changes in the signalling network induced by LPS using CellChat for analysing cell–cell communication [29]. This analysis revealed enhanced macrophage–macrophage signalling (Fig. 1F) and upregulation of the nicotinamide phosphoribosyltransferase (NAMPT) pathway in macrophages after LPS stimulation (Fig. 1G). We further confirmed LPS-induced upregulation of CD38 and NAMPT in sorted macrophages through flow cytometry analysis (Supplementary Fig. S1C and D). As NAMPT is a crucial enzyme in the NAD salvage pathway following CD38-induced NAD degradation [30, 31], these findings suggest that upregulation of *Cd38* expression induced by LPS may influence cell–cell signalling in macrophages via NAD metabolism.

### CD38 ligation aggravated LPS-induced inflammation in the kidney

**Figure 2:**
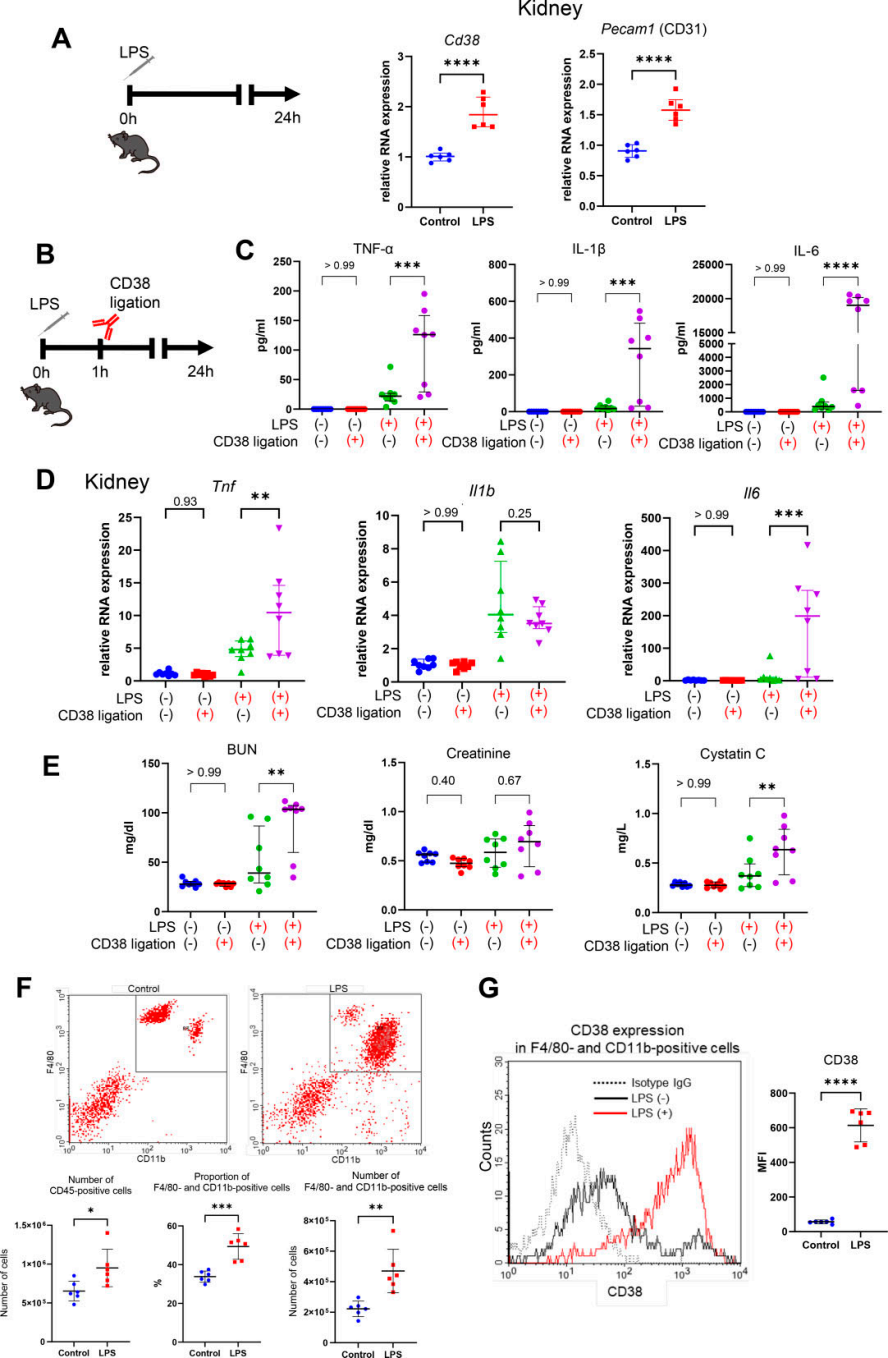
CD38 ligation aggravated LPS-induced inflammation response in the kidney. **(A)** Expression of *Cd38* and *Pecam1* mRNA in the kidney following LPS or vehicle injection (*n* = 8, unpaired *t*-test). **(B)** Schematic representation of the CD38 ligation experiment in the LPS-induced sepsis model. **(C)** Serum levels of TNF-α, IL-1β and IL-6 in four groups stratified by LPS and CD38 ligation (*n* = 8, Kruskal–Wallis test with Dunn's multiple comparison test). **(D)** Expression of *Tnf*, *Il1b* and *Il6* mRNAs in the kidney following CD38 ligation or isotype control injection (*n* = 8, unpaired *t*-test or Wilcoxon signed-rank test). **(E)** Serum levels of urea nitrogen, creatinine and cystatin C in four groups stratified by LPS and CD38 ligation (*n* = 8, Kruskal–Wallis test with Dunn's multiple comparison test). **(F)** Evaluation of F4/80 and CD11b expression in renal CD45-positive cells following LPS or vehicle injection (*n* = 6, unpaired *t*-test). **(G)** Assessment of CD38 expression in renal F4/80- and CD11b-positive macrophages following LPS or vehicle injection (*n* = 6, unpaired *t*-test). *P < .05, **P < .01, ***P < .001, ****P < .0001.

We investigated *Cd38* expression in the various organs of sepsis-induced mice after LPS exposure (Fig. 2A, Supplementary Fig. S3A). Both *Cd38* and *Pecam1* (CD31), encoding ligands of CD38, were upregulated in the kidney, brain, heart, spleen and intestine. Subsequently, CD38 ligation was induced by administering a well-established agonistic anti-CD38 antibody (clone NIMR-5) to the LPS-induced sepsis model (Fig. 2B). Notably, CD38 ligation significantly increased serum TNF-α, IL-1β and IL-6 levels (Fig. 2C). To identify the organs responsible for cytokine production, we compared the mRNA expression levels of these inflammatory cytokines in each organ. Our findings revealed that upregulation of *Il6* expression was most prominent in the kidney, which increased >100 times upon CD38 ligation (Fig. 2D, Supplementary Fig. S3B). Additionally, CD38 ligation worsened the LPS-induced kidney dysfunction, as shown by elevated blood urea nitrogen and serum cystatin C levels (Fig. 2E). As CD38 ligation alone did not affect cytokine production or kidney function, we hypothesized that LPS might enhance the effect of CD38 ligation by upregulating *Cd38* expression in renal macrophages. To validate this, we performed flow cytometry analysis of isolated renal CD45-positive cells and observed that LPS increased the number of CD11b- and F4/80-positive macrophages in the kidney (Fig. 2F) and enhanced *Cd38* expression in macrophages (Fig. 2G). We also confirmed increased renal *Il6* production following CD38 ligation in an additional mouse model of sepsis induced by faecal suspension intraperitoneal injection [32] (Supplementary Fig. S3C). Therefore, the kidney is particularly susceptible to the effects of CD38 ligation in sepsis and may be a primary source of IL-6 production.

### Kidney snRNA-seq revealed *Il6* expression in stromal cells

**Figure 3:**
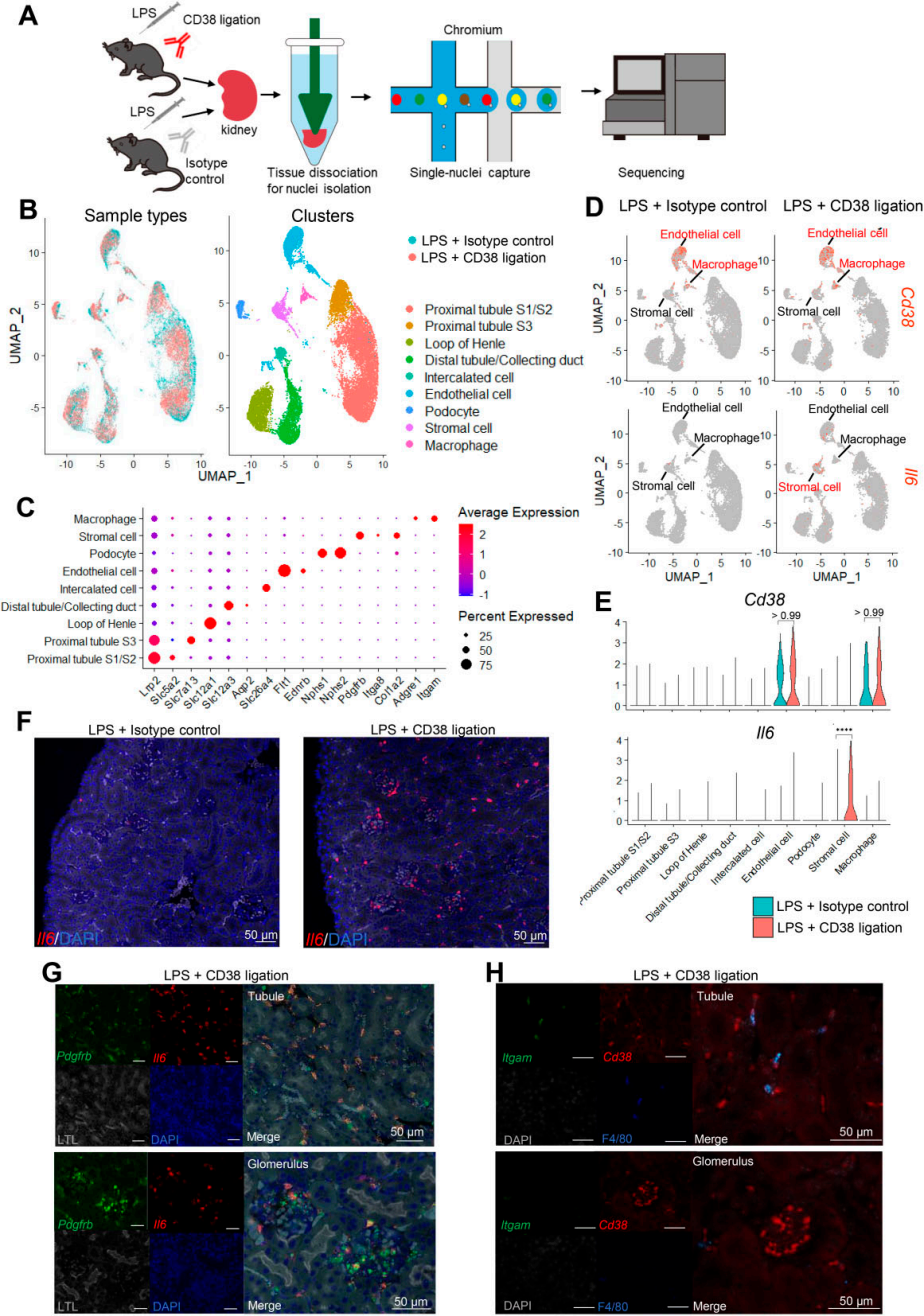
Kidney snRNA-seq analysis revealed *Il6* expression in stromal cells. **(A)** Schematic representation of the snRNA-seq protocol. **(B)** Uniform manifold approximation and projection plot displaying cell clusters in the kidney. **(C)** Expression of cell type–specific markers used for cluster identification presented as a dot plot. **(D)** Feature plot and **(E)** violin plot of *Cd38* and *Il6* expression. **(F)** ISH of *Il6* in the kidney following LPS injection with CD38 ligation or isotype control injection. **(G)** ISH of *Il6* and *Pdgfrb* in the kidney following LPS injection with CD38 ligation. **(H)** ISH of *Cd38* and *Itgam* with immunostaining of the macrophage marker F4/80 in the kidney following LPS injection with CD38 ligation. In ISH analysis, the tissue sections were evaluated in a minimum of 10 randomly selected high-power fields per sample.

To elucidate the mechanism underlying IL-6 production in the kidney, we performed snRNA-seq on kidney tissues collected after CD38 ligation (Fig. 3A). We identified nine clusters: proximal tubule S1/2 segment cells, proximal tubule S3 segment cells, loops of Henle cells, distal tubule/collecting duct cells, intercalated cells, podocytes, endothelial cells, stromal cells and macrophages (Fig. 3B and C). Concurrent with the increased circulating levels of inflammatory cytokines (Fig. 2C), we observed a notable upregulation of inflammation- and immune response–related genes in various tubular cells and podocytes owing to CD38 ligation (Supplementary Fig. S4). Following CD38 ligation, *Cd38* and *Pecam1* (CD31) expression was observed in macrophages and endothelial cells, whereas *Il6* was predominantly expressed in stromal cells, although these stromal cells rarely exhibited *Cd38* expression (Fig. 3D and E, Supplementary Fig. S5). Three clusters of stromal cells were identified, including fibroblasts, mesangial cells and pericytes (Supplementary Fig. S6A and B). Notably, the upregulation of *Il6* was observed in all three clusters (Supplementary Fig. S6C). ISH analysis revealed a significant upregulation of *Il6* in the interstitial and glomerular cells induced by CD38 ligation (Fig. 3F). Multiple ISH experiments demonstrated the co-expression of *Il6* and *Pdgfrb*, a gene specifically expressed in renal stromal cells [33, 34] (Fig. 3G). Conversely, *Il6* was not expressed in CD11b- and CD38-positive macrophages (Supplementary Fig. S7). *Pdgfrb*-positive cells in the interstitial and glomerular areas were mainly identified as fibroblasts and mesangial cells, respectively [34, 35]. *Cd38*-, *Itgam*- and F4/80-positive macrophages were predominantly located in the interstitial areas (Fig. 3H). These findings indicate the signalling interactions between CD38-positive macrophages and stromal cells, particularly within the renal interstitial area.

### NAMPT signalling was upregulated by CD38 ligation between macrophages and stromal cells

**Figure 4:**
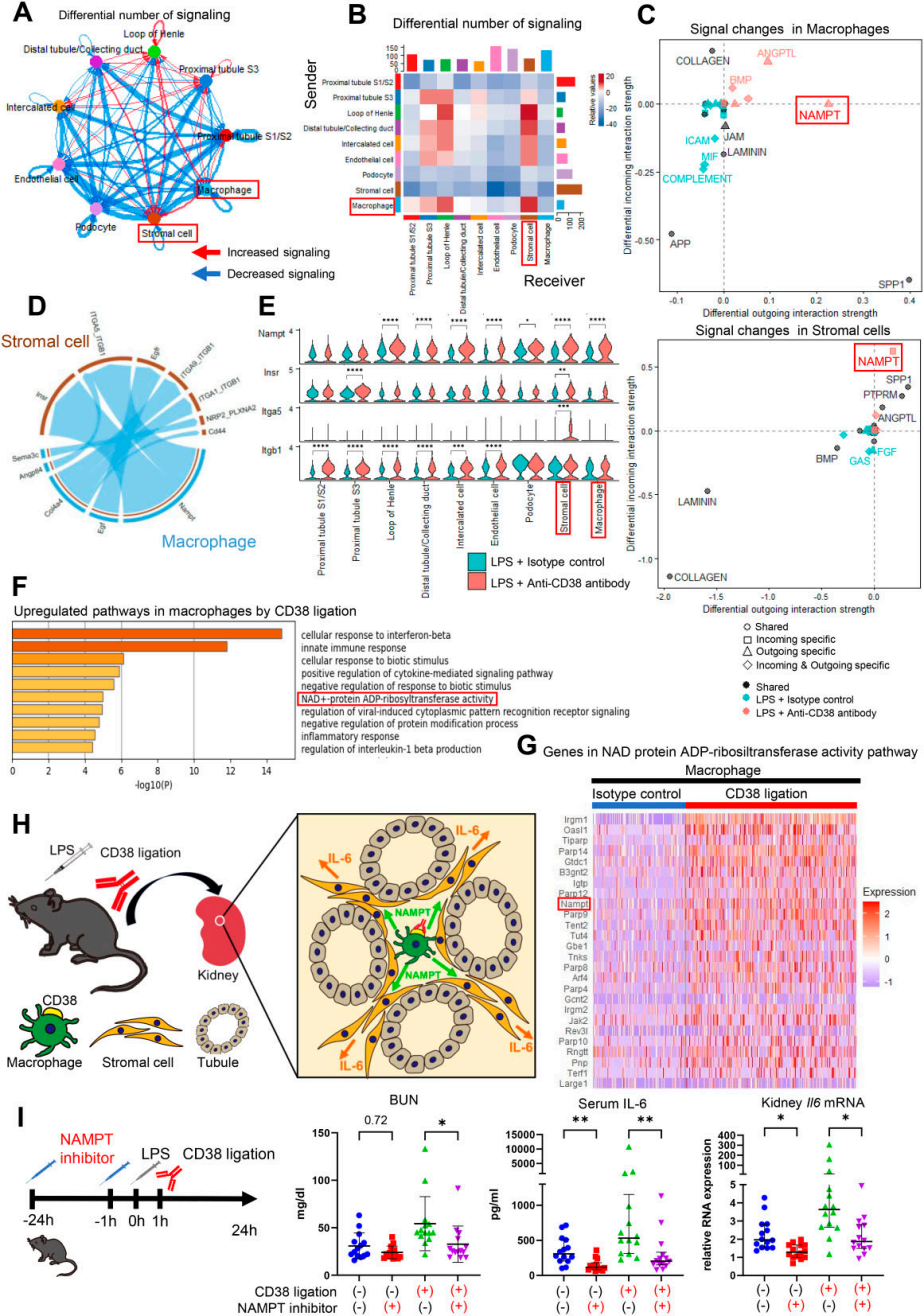
NAMPT signalling between macrophages and stromal cells was upregulated by CD38 ligation. The differential number of interactions in the cell–cell communication network was visualized using **(A)** a circle plot and **(B)** a heat map. **(C)** Specific signalling changes in macrophages and stromal cells after CD38 ligation visualized using a scatter plot. **(D)** Chord diagram showing the ligand receptors or signalling pathways upregulated by CD38 ligation from macrophages to stromal cells. **(E)** Violin plot showing genes involved in the NAMPT pathway. **(F)** Enrichment analysis performed with significantly upregulated genes in macrophages following CD38 ligation. **(G)** Heat map showing expression of the NAD–protein ADP–ribosyltransferase activity pathway genes in macrophages. Genes that were significantly upregulated in macrophages after CD38 ligation were used for constructing a heat map. **(H)** Schematic representation of NAMPT signalling between macrophages and stromal cells following CD38 ligation. **(I)** Blood urea nitrogen, serum IL-6 and kidney *Il6* expression following NAMPT inhibitor (FK866) injection in an LPS-induced sepsis model with or without CD38 ligation (*n* = 16, Kruskal–Wallis test with Dunn's multiple comparison test). *P < .05, **P < .01.

We performed cell–cell communication analysis using CellChat to identify the signalling pathways between CD38-positive macrophages and stromal cells and observed an increase in the number of differential interactions between the macrophages and stromal cells after CD38 ligation (Fig. 4A and B). Upon closer examination of specific signalling changes in each cell cluster, a significant upregulation of the outgoing signal of the macrophages and the incoming signal of the stromal cells was observed in the NAMPT pathway (Fig. 4C). The ligand–receptor pairs responsible for the upregulated signalling from the macrophages to the stromal cells are shown in Fig. 4D. *Nampt* expression was elevated in various cell types, including macrophages, whereas the receptors of NAMPT, *Insr* and *Itga5*, were specifically upregulated in the stromal cells (Fig. 4E). Enrichment analysis in macrophages demonstrated upregulation of the NAD metabolism pathway after CD38 ligation (Fig. 4F), with *Nampt* being among the significantly upregulated genes in this pathway (Fig. 4G). As NAMPT is a crucial enzyme required for NAD salvage after CD38-induced NAD degradation [30, 31], CD38 ligation may increase NAMPT expression by upregulating NAD metabolism. Additionally, NAMPT acts not only as an intracellular enzyme, but also as an inflammatory cytokine that induces IL-6 production [31, 36, 37]. Therefore, we hypothesized that the NAMPT pathway acts as a signalling route from CD38-ligated macrophages to stromal cells expressing IL-6 (Fig. 4H). To confirm this, we used an NAMPT inhibitor, FK866, in this sepsis model and observed that FK866 significantly attenuated kidney injury and IL-6 production induced by LPS and CD38 ligation (Fig. 4I). ISH analysis revealed that FK866 significantly reduced *Il6* expression in renal stromal cells (Supplementary Fig. S8). These findings indicate that the NAMPT pathway is involved in the exacerbation of sepsis induced by CD38 ligation.

### Histological analysis of human sepsis tissues revealed elevated expression of the *IL6* mRNA in renal stromal cells and NAMPT in renal CD38-positive macrophages

**Figure 5:**
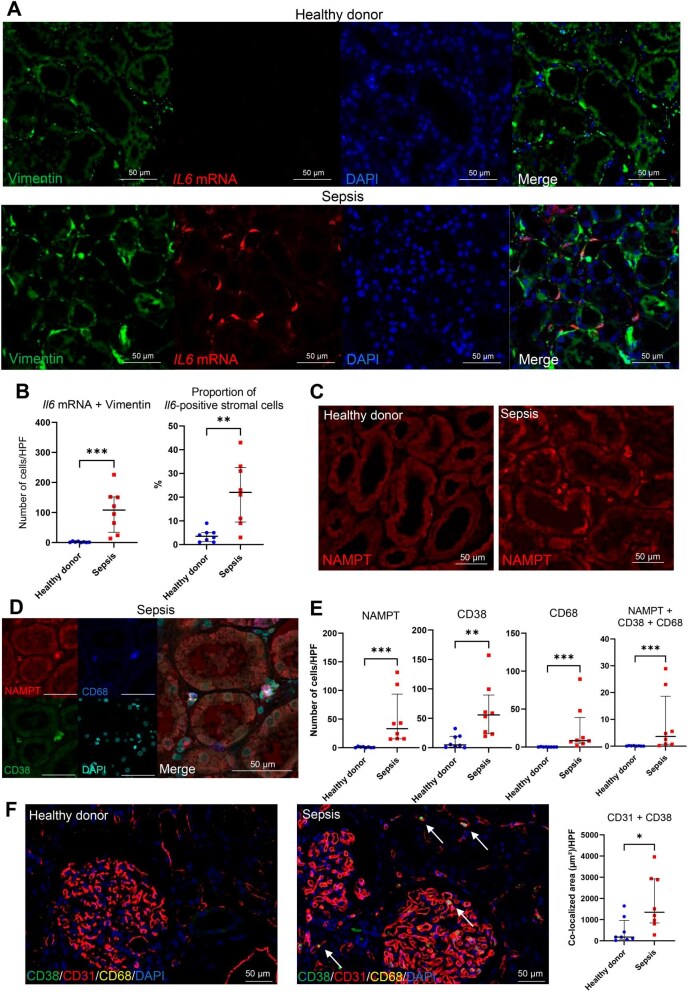
Histological analysis of human sepsis tissues revealed elevated expression of *IL6* mRNA in renal stromal cells and NAMPT in renal CD38-positive macrophages. **(A)**  *ISH* of *IL6* with immunostaining of the stromal cell marker vimentin. **(B)** The number of *IL6*- or vimentin-positive cells evaluated in a minimum of 10 randomly selected high-power fields per sample (*n* = 8, Wilcoxon signed-rank test). **(C)** Immunostaining of NAMPT. **(D)** Multiple immunostainings of CD38, CD68 and NAMPT. **(E)** The number of CD38-, NAMPT- or CD68-positive cells evaluated in a minimum of 10 randomly selected high-power fields per sample (*n* = 8, Wilcoxon signed-rank test). **(F)** Multiple immunostainings of CD31, CD38 and CD68. The co-stained area of CD31 and CD38 was evaluated in a minimum of 10 randomly selected high-power fields per sample (*n* = 8, Wilcoxon signed-rank test). *P < .05, **P < .01, ***P < .001.

Finally, we performed immunostaining and ISH analyses of human septic tissues (*n* = 8) (Supplementary Table S1). *IL6* mRNA expression was predominantly detected in the kidney, liver and spleen (Supplementary Fig. S9). *IL6* expressed in kidney tissue co-localized partially with the stromal cell marker vimentin [38] and the number of cells co-expressing *IL6* and vimentin was significantly greater in the septic samples than in the healthy donors (*n* = 8) (Fig. 5A and B, Supplementary Table S2). Additionally, immunostaining analysis showed that NAMPT expression increased in interstitial cells (Fig. 5C) and the number of CD38- and CD68-positive macrophages expressing NAMPT was significantly elevated in the septic samples (Fig. 5D and E). Furthermore, the area of CD38 and CD31 co-localization was significantly greater in the septic samples than in the healthy ones (Fig. 5F), which was indicative of increased CD38 ligation. Collectively, these results suggest that the CD38-related signalling observed in mice with sepsis is consistent with that observed in humans.

## DISCUSSION

This study elucidated the implications of CD38 ligation in sepsis and identified common signalling pathways in mice and humans with sepsis. LPS upregulated the expression of CD38 in peritoneal and renal macrophages and CD38 ligation significantly exacerbated inflammation, particularly in the kidneys. Kidney snRNA-seq analysis revealed that CD38 ligation induced IL-6 production in renal stromal cells via NAMPT signalling from CD38-positive macrophages. Histological analysis of human septic tissues showed upregulation of *IL6* mRNA and NAMPT in renal stromal cells and CD38-positive macrophages, respectively.

This is the first study to reveal significant IL-6 production in the renal stromal cells of mice with CD38-ligated sepsis and humans with severe sepsis. To the best of our knowledge, renal stromal cells have never been considered the primary source of inflammatory cytokines in sepsis. Although they produce IL-6 in response to inflammatory factors [39, 40], they have not been comprehensively investigated in mice or humans under conditions of sepsis. As the circulating level of IL-6 correlates highly with the outcome of patients with sepsis [41], identifying the supply source of IL-6 and elucidating its mechanism may improve our understanding and treatment of sepsis. This study revealed a novel role for renal stromal cells in the acute inflammation of sepsis, positioning them as potential therapeutic targets.

NAMPT has been identified as a signalling mediator between CD38-positive macrophages and stromal cells. NAMPT, the rate-limiting enzyme in the NAD biosynthetic pathway, modulates macrophage function by controlling intracellular NAD concentrations [30, 31]. A decrease in intracellular NAD levels reportedly upregulates NAMPT expression to maintain intracellular NAD levels, which can be restored by supplementation with an NAD precursor [42]. As CD38 is one of the main NAD-degrading enzymes, CD38 ligation may upregulate NAMPT expression by promoting NAD consumption. LPS also reduces intracellular NAD levels in macrophages by upregulating CD38 [43]. Therefore, the combination of LPS and CD38 ligation may induce significant NAD depletion in macrophages and increase NAMPT expression. Considering that supplementation with NAD precursors ameliorates LPS-induced inflammation [15, 44], the NAMPT pathway identified in this study may be a novel factor in NAD-associated inflammatory modulation.

Extracellular NAMPT induces the production of inflammatory cytokines, including TNF-α, IL-1β and IL-6, in peripheral monocytes and synovial fibroblasts [36, 37]. Hence we considered that the excessive NAMPT production induced by LPS administration and CD38 ligation in macrophages induced the extracellular release of NAMPT, promoting IL-6 production in the stromal cells (Fig. 4H). Although NAMPT is upregulated in other cell types, inflammatory cytokines, including IL-6, are known to upregulate NAMPT expression [45, 46]. Consequently, elevated levels of circulating inflammatory cytokines may secondarily upregulate NAMPT expression in various cell types. As NAMPT inhibition significantly ameliorated IL-6 production and kidney injury in this study, NAMPT is not merely a secondarily upregulated protein, but is also an essential factor in LPS- and CD38 ligation–induced inflammation.

This study, for the first time, presents evidence regarding elevated NAMPT expression in the kidneys of patients with sepsis. Notably, previous studies have linked the serum level of NAMPT to the prognosis of patients with sepsis [47]. In addition, previous studies have indicated that NAMPT inhibition by FK866 can alleviate sepsis-induced neuroinflammation and lung injury in mouse models [48, 49]. However, the origin and targets of NAMPT in sepsis have not yet been elucidated. This study uncovered the NAMPT signalling pathway from the CD38-positive macrophages to the renal stromal cells in a mouse model of sepsis and validated these findings in human samples. Additionally, we observed that NAMPT inhibition not only improved kidney injury, but also reduced circulating IL-6 levels. Therefore, NAMPT signalling may be a novel therapeutic target for sepsis-induced kidney injury and systemic inflammation. Given that several NAMPT inhibitors have been developed as anti-cancer agents [50], we propose that these inhibitors could potentially be repurposed for the treatment of sepsis.

Our study also has a few limitations that must be considered when interpreting the results. First, the mechanism by which CD38 ligation induces NAMPT expression remains unclear. Although we have explained the NAD metabolism, further investigation is required to validate the metabolic changes in NAD induced by CD38 ligation. Second, the reason underlying the elevation in serum TNF-α and IL-1β levels after CD38 ligation was not clarified in this study. Although NAMPT is known to induce these cytokines [36, 37], we did not observe significant upregulation of these cytokines in the organs investigated. Therefore, further studies are required to elucidate these underlying mechanisms. Third, CD31 expression in human kidney tissue is widespread, making it challenging to differentiate between circulating and locally expressed CD31. Further research using human samples is necessary to clarify the involvement of circulating CD31 in CD38 ligation during sepsis. Lastly, the CD38–NAMPT pathway may not be restricted to macrophages and stromal cells. Although macrophage–stromal cell crosstalk was predominant in our study, CD38 ligation may also have significant effects on other cell types, such as endothelial cells, in a unidirectional manner.

In conclusion, we uncovered the implications of CD38 ligation in an LPS-induced sepsis model and identified shared signalling pathways in both mice and humans with sepsis. The elevated expression of *Il6* mRNA in the renal stromal cells and increased NAMPT expression in CD38-positive macrophages represent novel findings in both mice and humans with sepsis. These findings provide novel therapeutic targets for the treatment of systemic inflammation and kidney injury induced by severe sepsis.

## Supplementary Material

gfae269_Supplemental_Files

## Data Availability

The sequence datasets produced in this study were deposited in the DDBJ Sequenced Read Archive and can be accessed using the following URL: https://www.ddbj.nig.ac.jp/dra/index-e.html. The accession numbers for the scRNA-seq of mouse peritoneal cells are DRR518117–DRR518119 and for the snRNA-seq of mouse kidney are DRR518120 and DRR518121.
