# Supplementary material for: CD38 ligation in sepsis promotes nicotinamide phosphoribosyltransferase-mediated IL-6 production in kidney stromal cells
Source: Nephrol Dial Transplant. 2024 Nov 20;40(7):1310–21. doi: 10.1093/ndt/gfae269 (PMC12207605; doi:10.1093/ndt/gfae269)
Supplement: gfae269_Supplemental_Files [file gfae269_supplemental_files.zip › Supplementary_figures.pdf]

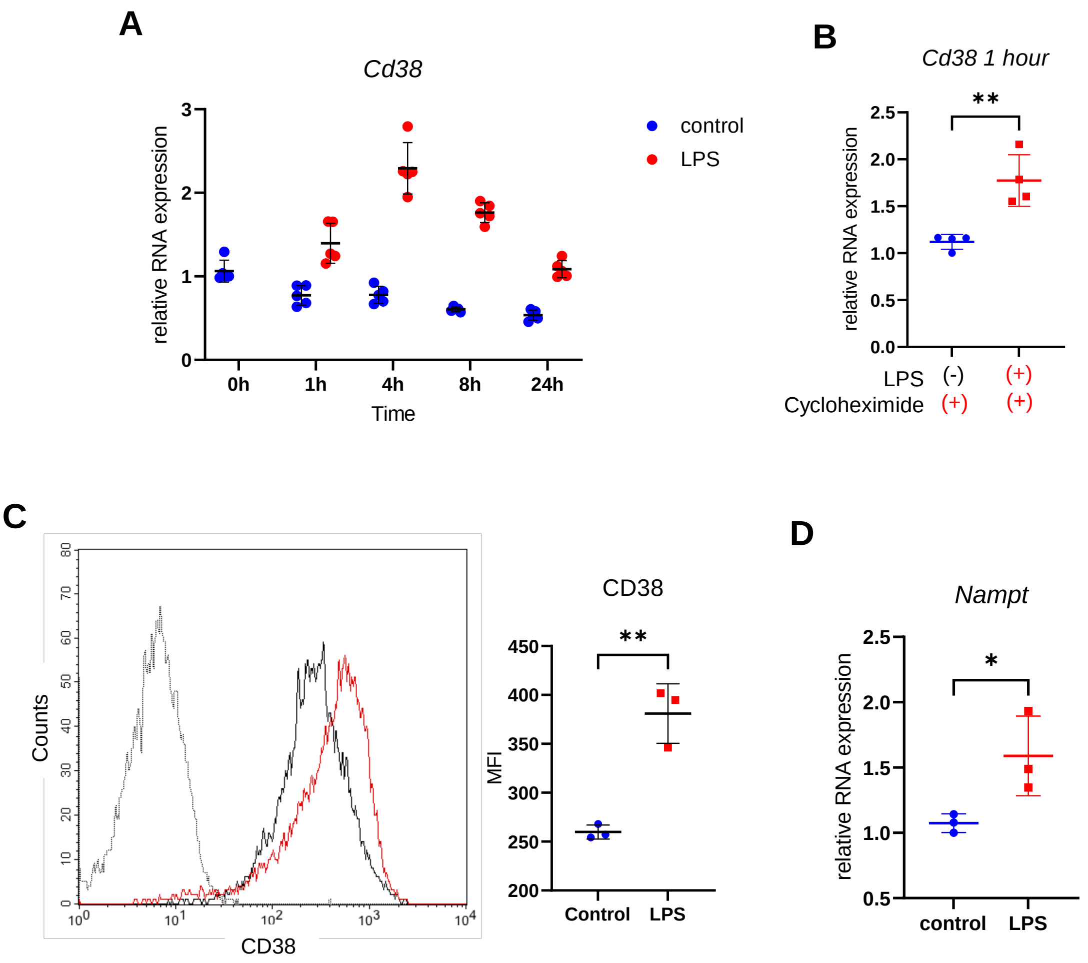

Supplemental Figure S1. Lipopolysaccharide (LPS) stimulation directly increased Cd38 expression in mouse peritoneal cells. (A) Expression of *Cd38* mRNA in mouse peritoneal cells following LPS stimulation (n = 5). **(B)** Expression of *Cd38* mRNA in mouse peritoneal macrophages following LPS stimulation under protein synthesis inhibition by cycloheximide (n = 4, unpaired t-test). **(C)** Assessment of CD38 expression in peritoneal F4/80- and CD11b-positive macrophages following LPS stimulation (n = 3, unpaired t-test). **(D)** Expression of *Nampt* mRNA in peritoneal F4/80- and CD11b-positive macrophages following LPS stimulation (n = 3, unpaired t-test). *P < 0.05, **P < 0.01.

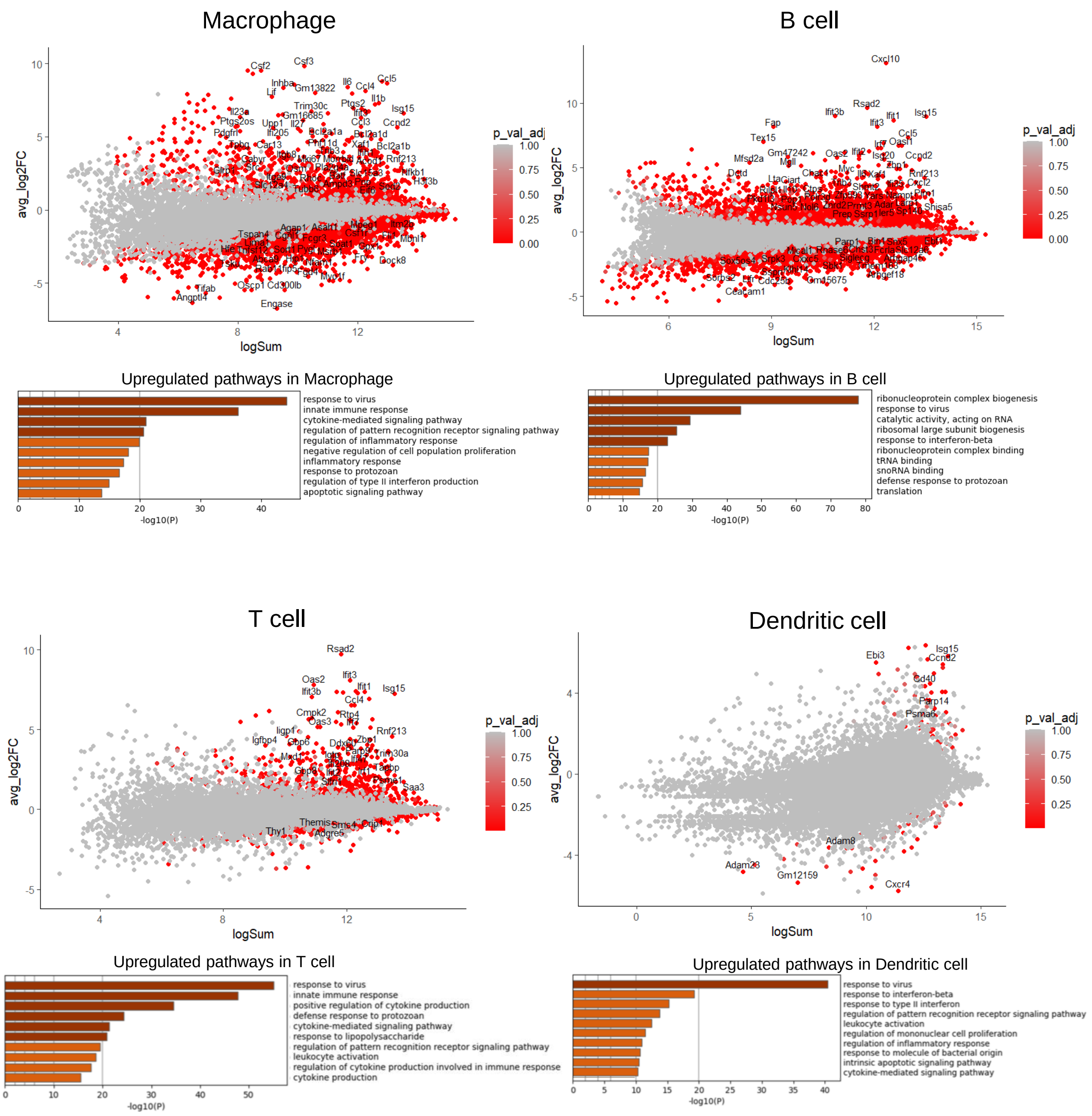

Supplemental Figure S2. Lipopolysaccharide (LPS) stimulation upregulated innate immunity-related genes. The MA plot generated with all detected genes, and the bar plot representing the enrichment analysis of gene ontology biological processes. Enrichment analysis was conducted using the significantly upregulated genes induced by LPS stimulation in each cluster.

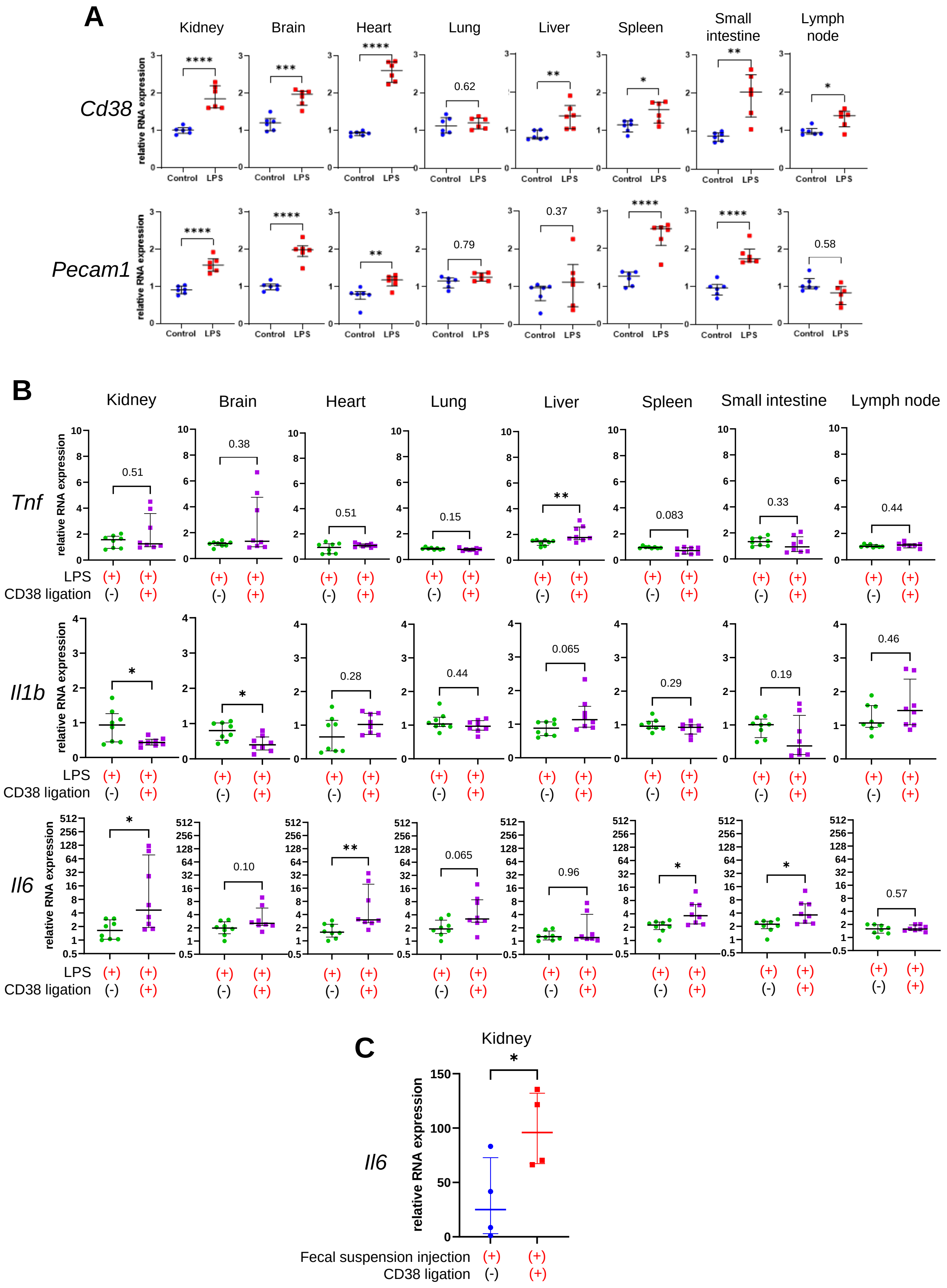

A
Kidney
Brain
Heart
Lung
Liver
Spleen
Small intestine
Lymph node
Cd38
Pecam1
relative RNA expression
Control
LPS
****
***
****
0.62
**
*
**
*
****
****
**
0.79
0.37
****
****
0.58
B
Tnf
Il1b
Il6
0.51
0.38
0.51
0.15
**
0.083
0.33
0.44
*
*
0.28
0.44
0.065
0.29
0.19
0.46
*
0.10
**
0.065
0.96
*
*
0.57
LPS
CD38 ligation
(+)
(-)
C
Kidney
Il6
relative RNA expression
*
Fecal suspension injection
CD38 ligation

Supplemental Figure S3. Inflammatory response in each organ in septic condition. (A) Expression of *Cd38* and *Pecam1* mRNA in each organ following LPS or vehicle injection (n = 8, unpaired t-test). **(B)** Expression of *Tnf*, *Il1b*, and *Il6* mRNAs in each organ following CD38 ligation or isotype control injection (n = 8, unpaired t-test or Wilcoxon signed-rank test). **(C)** Expression of *Il6* mRNA following fecal suspension injection and CD38 ligation. (n = 4, unpaired t-test). *P < 0.05, **P < 0.01, ***P < 0.001, ****P < 0.0001.

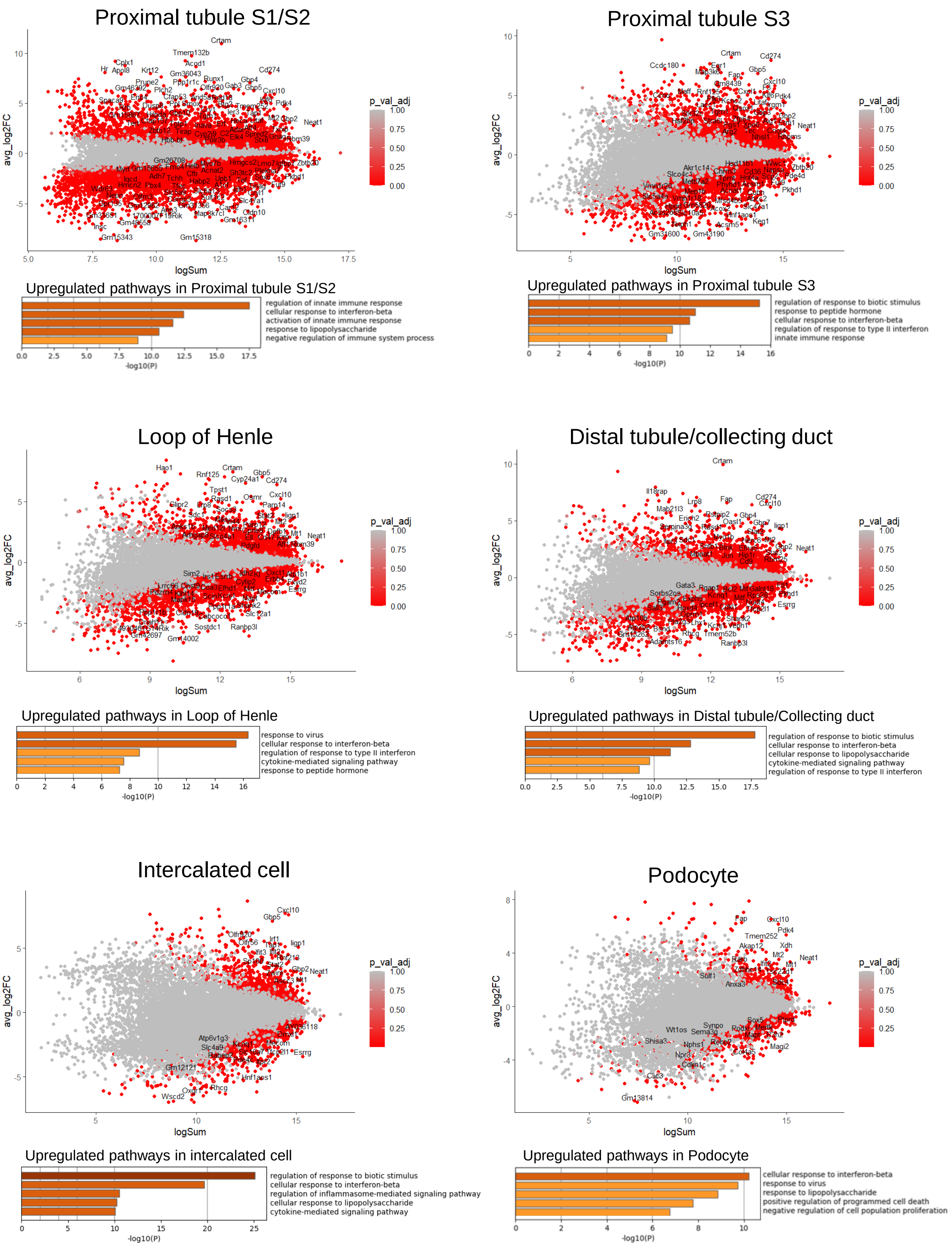

Proximal tubule S1/S2
avg_log2FC
logSum
p_val_adj
1.00
0.75
0.50
0.25
0.00
Upregulated pathways in Proximal tubule S1/S2
regulation of innate immune response
cellular response to interferon-beta
activation of innate immune response
response to lipopolysaccharide
negative regulation of immune system process
-log10(P)
Proximal tubule S3
Upregulated pathways in Proximal tubule S3
regulation of response to biotic stimulus
response to peptide hormone
cellular response to interferon-beta
regulation of response to type II interferon
innate immune response
Loop of Henle
Upregulated pathways in Loop of Henle
response to virus
cellular response to interferon-beta
regulation of response to type II interferon
cytokine-mediated signaling pathway
response to peptide hormone
Distal tubule/collecting duct
Upregulated pathways in Distal tubule/Collecting duct
regulation of response to biotic stimulus
cellular response to interferon-beta
cellular response to lipopolysaccharide
cytokine-mediated signaling pathway
regulation of response to type II interferon
Intercalated cell
Upregulated pathways in intercalated cell
regulation of response to biotic stimulus
cellular response to interferon-beta
regulation of inflammasome-mediated signaling pathway
cellular response to lipopolysaccharide
cytokine-mediated signaling pathway
Podocyte
Upregulated pathways in Podocyte
cellular response to interferon-beta
response to virus
response to lipopolysaccharide
positive regulation of programmed cell death
negative regulation of cell population proliferation

Supplemental Figure S4. CD38 ligation upregulated inflammation- and immune response-related genes in various tubular cells and podocytes. The MA plot generated with all detected genes, and the bar plot representing the enrichment analysis of gene ontology biological processes in renal tubular cells and podocytes. Enrichment analysis was conducted using the significantly upregulated genes induced by CD38 ligation in each cluster.

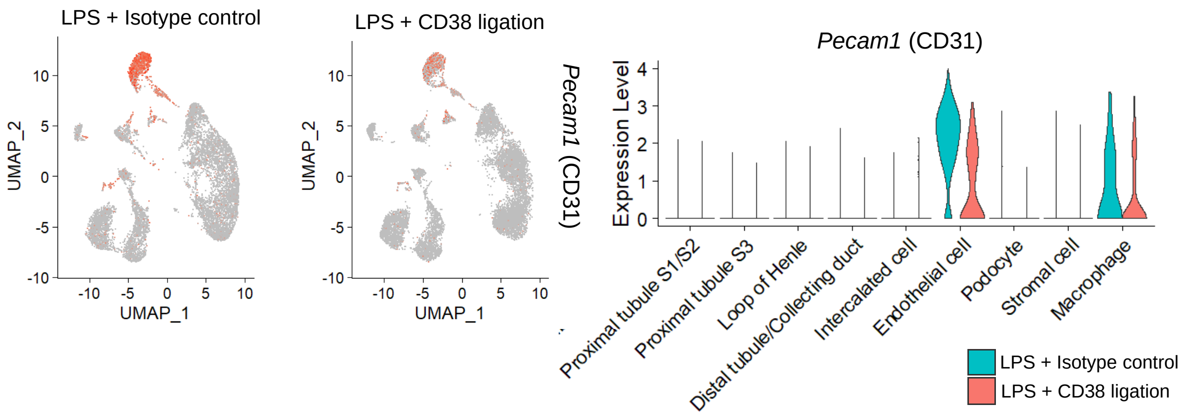

Supplemental Figure S5. *Pecam1* (CD31) was expressed in both endothelial cells and macrophages. Feature plot and violin plot of *Pecam1* (CD31) expression.

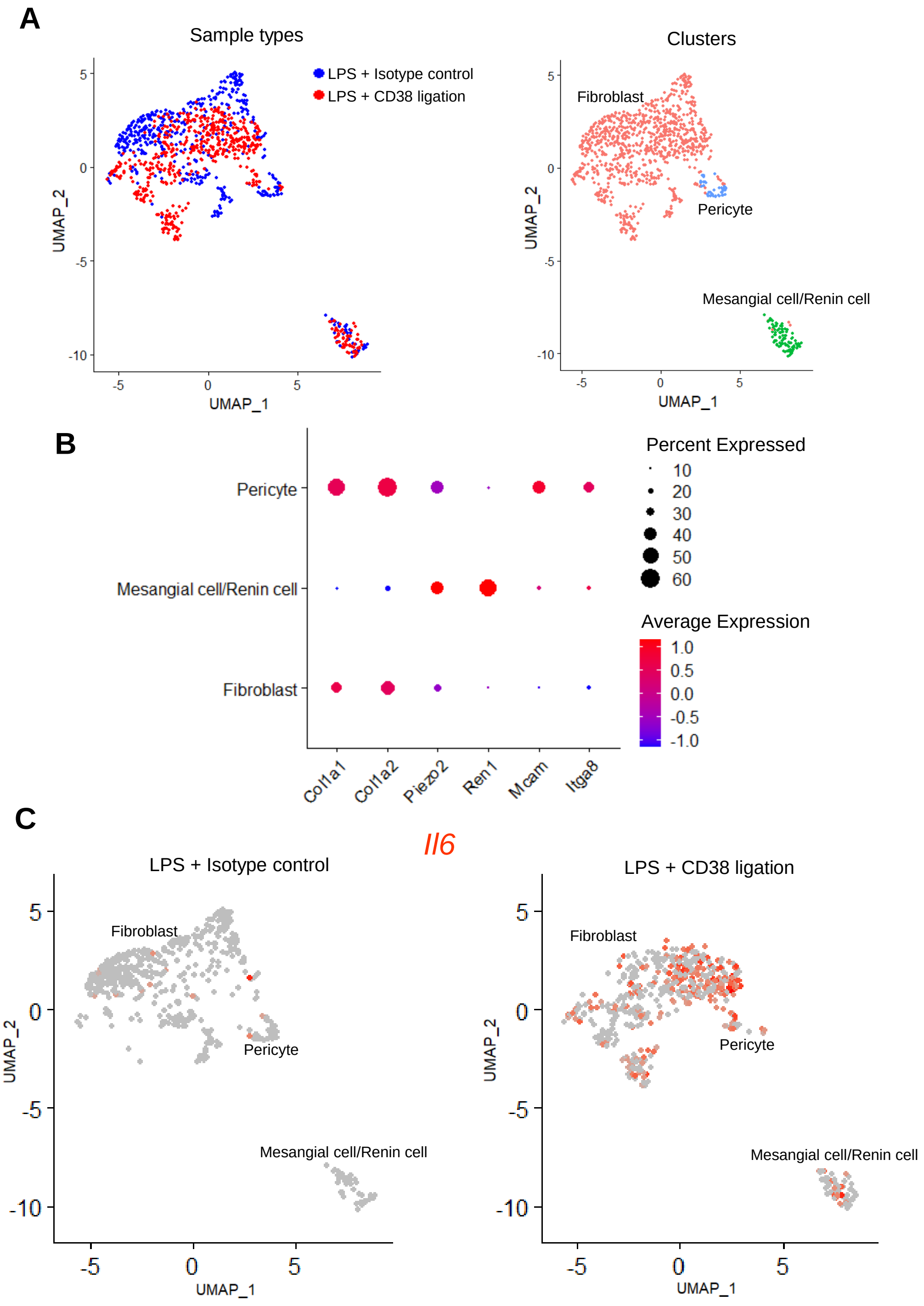

Supplemental Figure S6. All cell types within the stromal cell cluster expressed *Il6* following CD38 ligation. (A) Uniform manifold approximation and projection (UMAP) plot displaying cell clusters. **(B)** Dot plot presenting the expression of cell type–specific markers used for cluster identification. **(C)** Feature plot illustrating *Il6* expression.

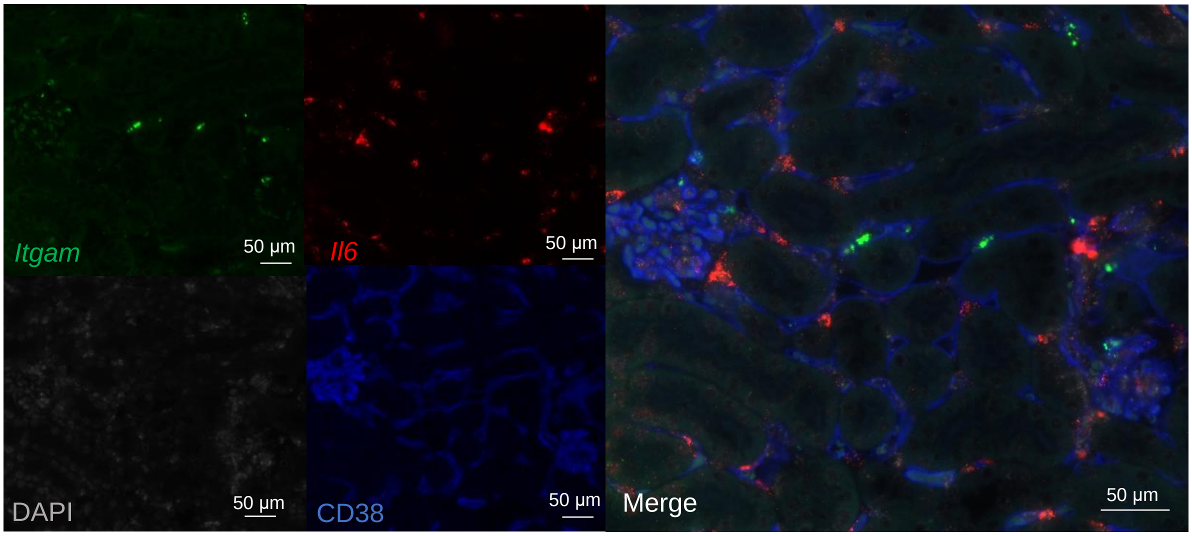

Supplemental Figure S7. *Il6* mRNA staining did not colocalize with *Itgam* mRNA and CD38 protein. *In situ* hybridization of *IL6* and *Itgam* with immunostaining of CD38.

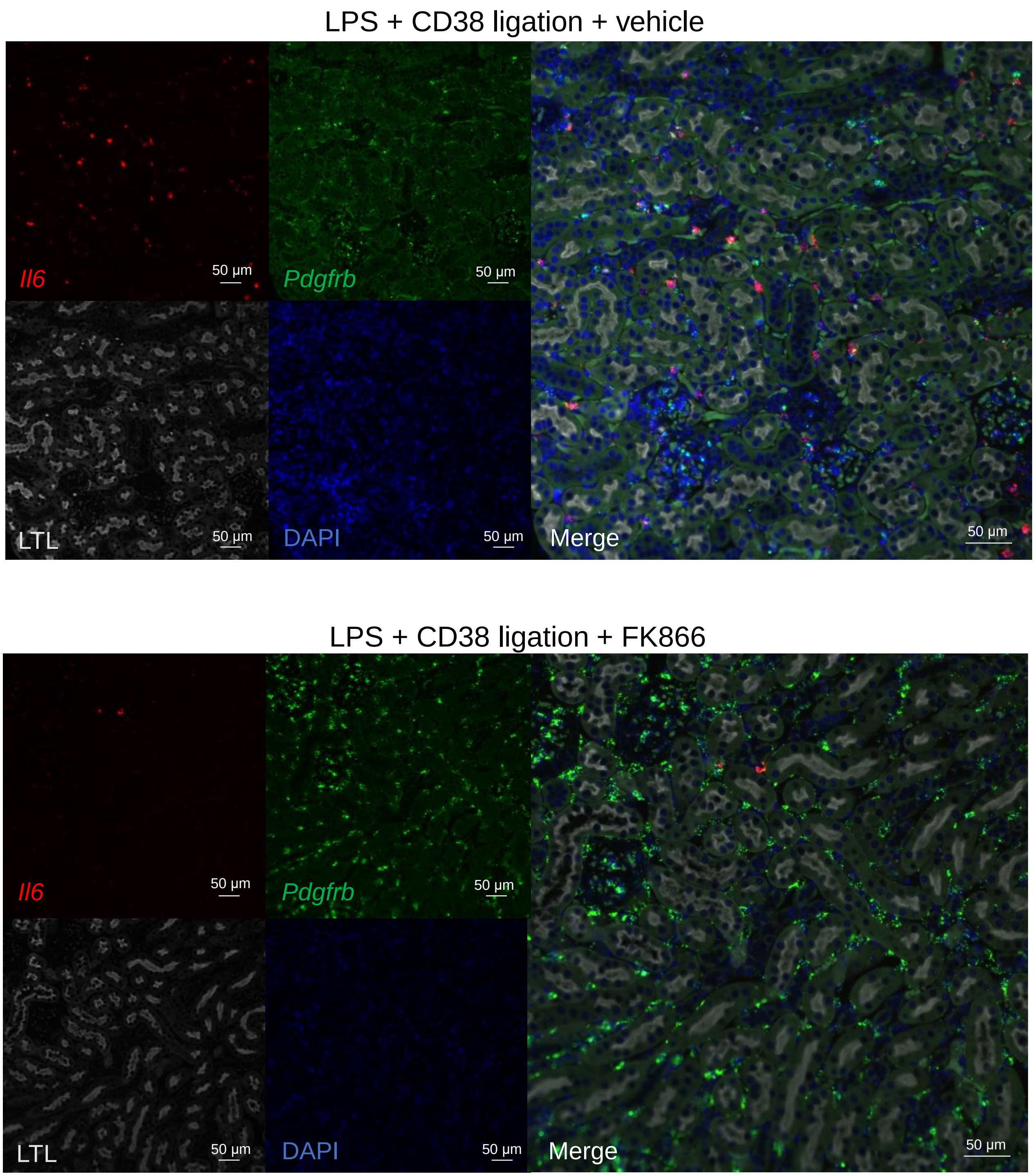

Supplemental Figure S8. NAMPT inhibitor (FK866) reduced *Il6* expression in stromal cells. *In situ* hybridization of *Il6* in the kidney following LPS injection and CD38 ligation, with or without NAMPT inhibitor FK866.

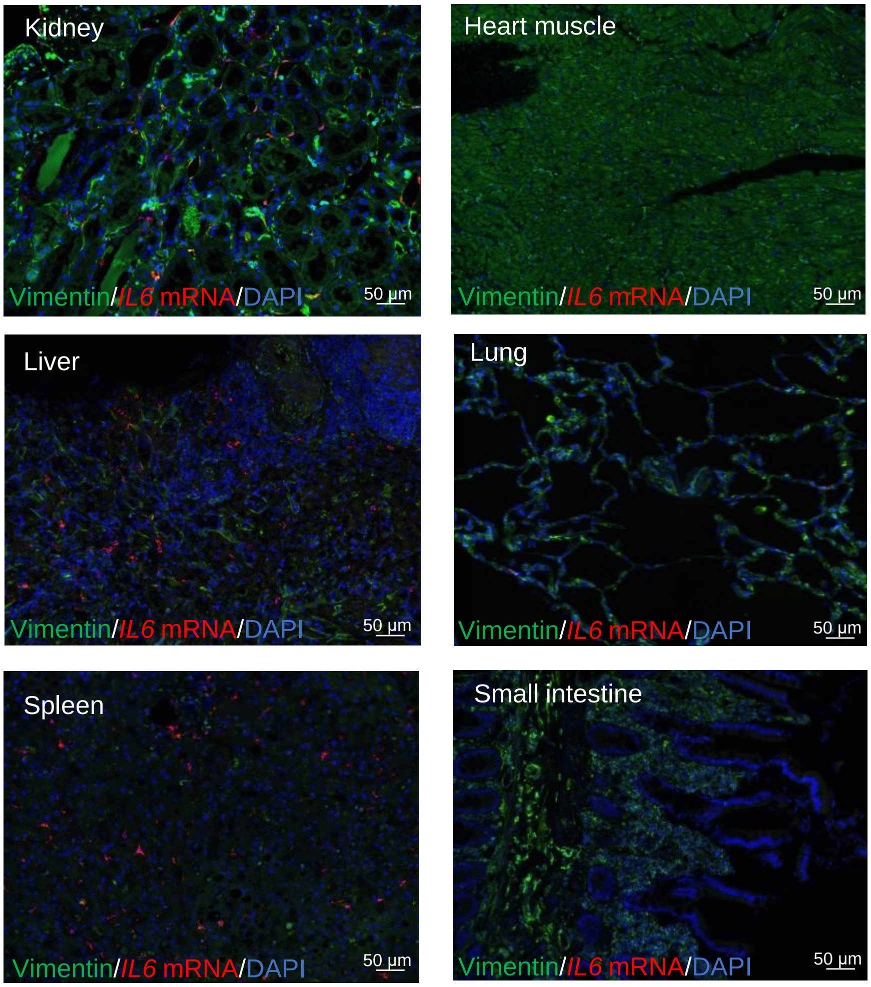

Supplemental Figure S9. Several organs including kidney expressed *IL6* mRNA in humans with sepsis. *In situ* hybridization of *IL6* performed in conjunction with immunostaining for Vimentin in the kidneys, heart muscle, lungs, liver, spleen, and small intestine of human subjects with sepsis.
