# Supplementary material for: CD38 ligation in sepsis promotes nicotinamide phosphoribosyltransferase-mediated IL-6 production in kidney stromal cells
Source: Nephrol Dial Transplant. 2024 Nov 20;40(7):1310–21. doi: 10.1093/ndt/gfae269 (PMC12207605; doi:10.1093/ndt/gfae269)
Supplement: gfae269_Supplemental_Files [file gfae269_supplemental_files.zip › Supplementary_methods.docx]

**Supplemental Materials and Methods**

**Animals**

C57 BL/6J mice were obtained from the Jackson Laboratory Japan (Kanagawa, Japan). To develop an lipopolysaccharide (LPS)-induced sepsis model, 8–12-week-old male C57 BL/6J mice were intraperitoneally injected with 10 mg/kg LPS from *Escherichia coli* O127:B8 (Sigma-Aldrich, St. Louis, MO, USA). The control group received vehicle injections only. Twenty-four hours after the LPS injection, the mice were intraperitoneally administered a three-anesthetic mixture of medetomidine, midazolam, and butorphanol before sacrifice. Blood and organ samples were collected under anesthesia for subsequent analyses. To establish a sepsis model via fecal suspension injection [32], 8–12-week-old male C57BL/6J mice were intraperitoneally injected with 15 mg of feces suspended in 300 µl of normal saline. The control group received only normal saline injections. Kidney samples were harvested 24 hours post-injection under anesthesia. For CD38 ligation, 50 mg/kg of agonistic anti-CD38 antibody (clone NIMR-5, Bio X Cell, Lebanon, NH, USA) or rat IgG2a isotype control (Bio X Cell) was injected intraperitoneally 1 h after the LPS or fecal suspension injection. The nicotinamide phosphoribosyltransferase (NAMPT) inhibitor, FK866 (Sigma-Aldrich), was administered intraperitoneally at doses of 20 mg/kg 24 and 1 h prior to the LPS injection. The serum levels of urea, creatinine, cystatin C, tumor necrosis factor-α (TNF-α), interleukin (IL)-1β, and IL-6 were measured using the QuantiChrom urease assay kit (BioAssay Systems, Hayward, CA, USA), Labassay creatinine kit (Fujifilm Wako chemicals, Osaka, Japan), cystatin C enzyme-linked immunosorbent assay (ELISA) kit (BioVendor, Heidelberg, Germany), Quantikine TNF-α ELISA kit (R&D Systems, Abingdon, UK), Quantikine IL-1β ELISA kit (R&D Systems), and Quantikine IL-6 ELISA kit (R&D Systems), respectively. All animal experiments were performed following the protocols approved by the Institutional Animal Care and Ethics Committee of Niigata University (approval no., SA01267 and SA01191).

**Human tissue samples**

Formalin-fixed sections of human organs, including the kidney, heart muscle, lungs, spleen, liver, and small intestine, were obtained from autopsy samples previously collected and stored at the Niigata University. Biopsy samples from renal transplantation donors were used as healthy controls. Sepsis samples were obtained from patients who underwent autopsies between 2000 and 2023, and were histologically diagnosed with sepsis. This study was approved by the Institutional Review Board of the Niigata University Graduate School of Medical and Dental Sciences, Niigata, Japan (approval no., 2023-0127), and was performed according to the principles of the Declaration of Helsinki. Informed consent was obtained in the form of opting out on the website (URL: https://www.med.niigata-u.ac.jp/contents/activity/clinical_research/kougen/index.html).

**Immunofluorescence staining with tyramide signal amplification**

For immunofluorescence analysis of CD38, CD68, and NAMPT expression in the human kidney, 4 μm-thick paraffin sections were stained with a rabbit monoclonal antibody specific for CD38 (catalog no.: ab108403, Abcam, Cambridge, UK; diluted 1:200 in phosphate-buffered saline [PBS]). Subsequently, the sections were incubated with N-Histofine® Simple Stain™ mouse MAX PO® (Rabbit) (Nichirei Biosciences, Tokyo, Japan), and the signals were detected using TSA Plus fluorescein (Akoya Biosciences, Menlo Park, CA, USA). Following tyramide detection, the sections were subjected to heat-mediated antigen retrieval and antibody stripping. They were subsequently stained with rabbit polyclonal antibodies specific for NAMPT (catalog no. 11776-1-AP, Proteintech, Rosemont, IL, USA; diluted 1:100 in PBS) and mouse monoclonal antibodies specific for CD68 (catalog no. ab955, Abcam; diluted 1:50 in PBS). They were then incubated with Alexa Fluor 596-conjugated anti-mouse IgG (Invitrogen, Carlsbad, CA, USA) and Cy5-conjugated anti-rabbit IgG (Abcam). Finally, the slides were mounted using ProLong Gold antifade mountant with NucBlue™ stain (Invitrogen). The number of CD38-, NAMPT-, or CD68-positive cells was evaluated in a minimum of 10 randomly selected high-power fields per sample using a BZ-X700 Analyzer ver.1.3 (Keyence, Osaka, Japan). For co-staining of CD38, CD31, and CD68 in the human kidney, the sections were subjected to tyramide signal amplification of CD38, as described above. Subsequently, the sections were subjected to heat-mediated antigen retrieval and antibody stripping and stained with a mouse monoclonal antibody specific to CD31 (catalog no.: ab9498, Abcam; diluted 1:50 in PBS). The sections were then incubated with N-Histofine® Simple Stain™ mouse MAX PO® (Mouse) (Nichirei Biosciences), and the signals were detected using TSA Plus cyanine 5 (Akoya Biosciences). Following the second tyramide detection, the sections were subjected to heat-mediated antigen retrieval and antibody stripping, followed by staining with a rabbit polyclonal antibody specific for CD68 (catalog no.: ab955, Abcam; diluted 1:50 in PBS). Finally, the sections were incubated with N-Histofine® Simple Stain™ mouse MAX PO® (Mouse) (Nichirei Biosciences), and the signals were detected using TSA Plus cyanine 3.5 (Akoya Biosciences). The co-stained areas of CD31 and CD38 were evaluated in a minimum of 10 randomly selected high-power fields per sample using a BZ-X700 Analyzer ver.1.3 (Keyence).

***In situ* hybridization**

The mouse kidney tissues were fixed in 10% neutral-buffered formalin and embedded in paraffin. Slides containing 4 μm-thick paraffin sections were subjected to *in situ* hybridization (ISH) using the RNAscope® multiplex fluorescent reagent kit v2 (Advanced Cell Diagnostics, Hayward, CA, USA), following the manufacturer's instructions. Briefly, tissue sections were incubated at 60 °C for 30 min in a HybEZTM II oven (Advanced Cell Diagnostics), pretreated with an H_2_O_2_ solution for 10 min, and boiled with RNAscope target retrieval for 15 min. The tissue sections were washed using 100% ethanol and incubated with Protease Plus for 30 min at 40 °C. Hybridization was performed using a combination of Probe-Mm-*Il6*-C1 and Probe-Mm-*Pdgfrb*-C2 for 2 h at 40 °C. After hybridization of the probes, the tissue sections were sequentially incubated in the ACD HybEZ™ II oven at 40 °C with amplification reagents: AMP-1 for 30 min, AMP-2 for 15 min, and AMP-3 for 30 min. The sections were then incubated with C1-horse radish peroxidase (HRP) at 40 °C for 15 min, and the hybridized signals were detected by incubating the sections with TSA Plus cyanine 3.5 (Akoya Biosciences). Following incubation with the HRP blocker at 40 °C for 15 min, incubation with C2-HRP and detection with TSA Plus Cyanine 5 were performed as mentioned above. Finally, the slides were mounted using ProLong Gold antifade mountant with NucBlue™ stain (Invitrogen).

***In situ* hybridization with immunofluorescence staining**

Four micrometer-thick paraffin sections of human tissues were subjected to ISH using Probe-Hs-*IL6*-C1, a combination of Probe-Mm-*Cd38*-C1 and Probe-Mm-*Itgam*-C2, or a combination of Probe-Mm-*Il6*-C1 and Probe-Mm-*Itgam*-C2 as described above. Subsequently, the sections were subjected to heat-mediated antigen retrieval and stained with a rabbit monoclonal antibody specific for vimentin (catalog no. 11776-1-AP; Proteintech; diluted 1:50 in PBS), a rat monoclonal antibody specific for F4/80 (catalog no. 50-4801-82; Invitrogen; diluted 1:100 in PBS) or a rat monoclonal antibody specific for CD38 (catalog no. ab216343; Abcam; diluted 1:400 in PBS). The sections were then incubated with Alexa Fluor 488-conjugated anti-rabbit IgG antibody (Invitrogen) or Cy5-conjugated anti-rat IgG antibody (Abcam). Finally, the slides were mounted using ProLong gold antifade mountant with NucBlue™ stain (Invitrogen). The number of *IL6*- or vimentin-positive cells was evaluated in a minimum of 10 randomly selected high-power fields per sample using the BZ-X700 Analyzer ver.1.3 (Keyence).

**Cell culture and LPS stimulation of mice peritoneal cells**

Peritoneal immune cells were collected *via* intraperitoneal injection of 10 mL PBS into 8–12-week-old male C57 BL/6J mice. The cells were centrifuged at 1,200 rpm for 4 min and resuspended in Dulbecco's modified Eagle's medium containing 10% heat-inactivated fetal bovine serum. Following an overnight incubation at 37 °C in a 5% CO_2_ cell culture incubator, the cells were exposed to 100 ng/mL LPS from *E. coli* O127:B8 (Sigma-Aldrich) for the indicated durations. Subsequently, the cells were collected and used for RNA extraction. For macrophage culture with a protein synthesis inhibitor, peritoneal macrophages were isolated from immune cells using the MACS cell separator and the Macrophage Isolation Kit (Miltenyi Biotec). The isolated macrophages were incubated overnight in Dulbecco's Modified Eagle's Medium containing 10% heat-inactivated fetal bovine serum. The following day, cells were treated with 1 µg/mL cycloheximide (Fujifilm Wako Chemicals) for 1 hour, followed by stimulation with 100 ng/mL LPS for 1 hour. Afterward, the cells were harvested for RNA extraction.

**Flow cytometry analysis**

For kidney single cell preparation, the kidneys were finely minced and digested using 0.4 mg/mL collagenase D (Roche, Basel, Switzerland) and 0.01 ng/mL DNase I in Roswell Park Memorial Institute-1640 medium supplemented with 10% heat-inactivated fetal bovine serum for 45 min at 37 °C. The cell suspensions were sequentially filtered through a 70-μm nylon mesh, resuspended in a 33% Percoll solution, and centrifuged at 2000 rpm for 20 min at room temperature. After red blood cell lysis, the kidney-infiltrating cells were incubated with CD45 microbeads (Miltenyi Biotec, Bergisch Gladbach, Germany) for 15 min and the CD45-positive cells were isolated using a MACS separator (Miltenyi Biotec, Biolegend, San Diego, CA, USA). The isolated CD45-positive cells were incubated with rat anti-mouse CD16/CD32 (clone 2.4G2) antibody to block nonspecific binding through FcγRs and subjected to flow cytometric analysis using fluorescein isothiocyanate-conjugated anti-mouse/human CD11b (clone M1/70, Biolegend), phycoerythrin-conjugated anti-mouse F4/80 (clone BM8, BioLegend), and allophycocyanin-conjugated anti-mouse CD38 (clone 90, BioLegend) antibody.

For flow cytometry analysis of peritoneal immune cells, cells were harvested from 8–12-week-old male C57BL/6J mice as described earlier. The cells were incubated with 100 ng/mL of LPS for 24 hours and subsequently stained with the same antibodies used for isolated CD45-positive renal cells. CD11b- and F4/80-positive cells were sorted as peritoneal macrophages using the FACSAria™ III Cell Sorter (BD Biosciences, San Jose, CA, USA) and then used for RNA extraction.

**Reverse transcription-quantitative polymerase chain reaction analysis**

Total RNA was extracted from mouse organs or cultured cells using the RNeasy mini kit (Qiagen, Hilden, Germany). Reverse transcription-quantitative polymerase chain reaction (RT-qPCR) analysis was performed using the Thermal Cycler Dice real-time system II with the One Step SYBR PrimeScript Plus RT-PCR kit (Takara Bio, Shiga, Japan), following the manufacturer's protocol. The target amplicons, *Gapdh*, and *Hprt1*, were reverse transcribed and quantified using the same template RNA for relative quantification. The primer sequences are listed in Supplemental Table 3.

**Single-cell RNA sequencing**

The mouse peritoneal immune cells were collected and cultured as described above. Cells were exposed to 100 ng/mL LPS from *E. coli* O127:B8 (Sigma-Aldrich) for 4 h and collected using cell scrapers. After passing through 40-µm cell strainers, single cells were captured using the Chromium System (10x Genomics, Pleasanton, CA, USA) according to the manufacturer's instructions. Briefly, the cells were washed using PBS containing 0.04% bovine serum albumin and loaded into a Chromium Next GEM Chip G with reagents of Chromium Next GEM single cell 3′ reagent kits v3.1 (10X Genomics). The cell-gel beads in the emulsion were generated and incubated to generate barcoded cDNA. The cDNA was cleaned using Dynabeads (10x Genomics) and amplified using 11 PCR cycles. The cDNA was then enzymatically fragmented, end-repaired, poly-A–tailed, adapter-ligated, and amplified using PCR. The constructed libraries were sequenced on an Illumina HiSeq 4000 platform (150 bp paired-end reads).

**Single-nucleus RNA sequencing**

Nuclei were isolated from frozen kidney tissue using a Chromium Nuclei Isolation Kit (10x Genomics), following the manufacturer's protocol. In brief, three samples from each group were selected, and one-quarter of the frozen kidney tissue was dissociated using pestles. The dissociated tissues were then mixed according to the groups and placed on a nuclear isolation column. Nuclei in the flow-through were washed using debris removal and wash buffers. Following passage through 40-µm cell strainers, the concentration of single nuclei was determined using 4′,6-diamidino-2-phenylindole staining. Single nuclei, diluted to a concentration of 1000 cells/µm, were captured using a Chromium System (10x Genomics) following the manufacturer's instructions.

**Analysis of single-cell and single-nucleus RNA sequencing**

The resulting FASTQ file was processed using Cell Ranger v7.1.0 (10x Genomics). The mouse genome (mm10) was used for alignment, and the CellRanger count was used for alignment and quantification of gene expression. The R software (version 4.3.0) was used for graphical and statistical analyses. The R package, Seurat (version 5.1.0), was used for data analysis, with exclusion criteria for cells with mitochondrial gene percentages exceeding 10% and unique gene counts below 1000.

For analyzing single-cell RNA sequencing (scRNA-seq) data from mouse peritoneal immune cells, integration of data from individual samples was performed using the Harmony package (version 1.1.0) post-normalization. Clustering was performed using four principal components from harmonized principal component analysis with a resolution of 0.1. Uniform manifold approximation and projection (UMAP) visualization was performed using four principal components from the harmonized principal component analysis. Differentially expressed genes (DEGs) between the clusters were identified using Seurat FindMarkers. Pathway enrichment analysis (Gene Ontology [GO] biological processes) was conducted on the DEGs using Metascape. Cell–cell communication analysis was performed using the CellChat package (version 1.6.1).

A similar approach was used for analyzing and integrating the single-nucleus RNA sequencing (snRNA-seq) data of the mouse kidney samples. The Harmony package (version 1.1.0) was used for data integration after normalization. Clustering was performed using 10 principal components from a harmonized principal component analysis with a resolution of 0.1. UMAP visualization was performed using 10 principal components from harmonized principal component analysis. DEGs between clusters were identified using Seurat's FindMarkers function after excluding clusters that predominantly consisted of doublet cells. Pathway enrichment analysis (GO biological processes) of DEGs was performed using Metascape. Cell–cell communication analysis was performed using the CellChat package (version 1.6.1).
