# Supplementary material for: CD38 ligation in sepsis promotes nicotinamide phosphoribosyltransferase-mediated IL-6 production in kidney stromal cells
Source: Nephrol Dial Transplant. 2024 Nov 20;40(7):1310–21. doi: 10.1093/ndt/gfae269 (PMC12207605; doi:10.1093/ndt/gfae269)
Supplement: gfae269_Supplemental_Files [file gfae269_supplemental_files.zip › Supplementary_tables.pdf]

Supplemental Table S1. Clinical Data of sepsis samples. The data on serum creatinine and BUN were measured at the last time point before death.

Sex	Age	Infection focus	Causative bacteria	Duration from onset to death (day)	Creatinine (mg/dl)	BUN (mg/dl)	Past history of CKD
Male	61	Unknown	*Streptococcus pneumoniae*	3	3.79	42	None
Male	67	Cholangitis	*Escherichia coli*	3	1.7	19	None
Male	56	Unknown	*Streptococcus pyogenic group*	2	3.32	50	None
Male	70	Pneumoniae	*Pseudomonas aeruginosa*	31	1.35	51	None
Female	44	Unknown	*Pseudomonas aeruginosa, Klebsiella pneumoniae*	2	1.38	38	None
Female	74	Cholangitis, Liver abscess	*Klebsiella oxytoca, Clostridium perfringes*	7	2.18	35	None
Female	75	Infectious endocarditis	*Coagulase(-) Staphylococcus*	23	1.2	20	None
Female	79	Cholangitis	*Citrobacter freundii, Klebsiella pneumoniae*	2	1.27	29	DKD (basal creatinine 1.0-1.1)

BUN, blood urea nitrogen; CKD, chronic kidney disease; DKD, diabetic kidney disease

Supplemental Table S2. Clinical Data of healthy donor samples. The data on serum creatinine and BUN were measured at the last time point before kidney transplantation.

Sex	Age	Creatinine (mg/dl)	BUN (mg/dl)
Female	46	0.54	9
Female	49	0.68	9
Female	47	0.67	10
Female	75	0.51	12
Female	61	0.62	13
Female	51	0.59	17
Female	69	0.53	12
Male	53	0.92	17

BUN, blood urea nitrogen

Supplemental Table S3. The primers used in reverse transcription-quantitative polymerase chain reaction (RT-qPCR).

Gene name	Sense (5'-3')	Antisense (3'-5')
Gapdh	AGGTCGGTGTGAACGGATTTG	GGGGTCGTTGATGGCAACA
Hprt1	TCAGTCAACGGGGGACATAAA	GGGGCTGTACTGCTTAACCAG
Cd38	TCTCTAGGAAAGCCCAGATCG	GTCCACACCAGGAGTGAGC
Pecam1	CCAAAGCCAGTAGCATCATGGTC	GGATGGTGAAGTTGGCTACAGG
Tnf	GGTGCCTATGTCTCAGCCTCTT	GCCATAGAACTGATGAGAGGGAG
Il1b	GAAATGCCACCTTTTGACAGTG	TGGATGCTCTCATCAGGACAG
Il6	CTGCAAGAGACTTCCATCCAG	AGTGGTATAGACAGGTCTGTTGG
Nampt	GGCACCACTAATCATCAGACCTG	AAGGTGGCAGCAACTTGTAGCC
